# A Retrospective Self-Controlled Study Evaluating the Prophylactic Effects of CACIPLIQ20 on Postsurgical Scars

**DOI:** 10.1093/asjof/ojad031

**Published:** 2023-03-23

**Authors:** Gilbert Zakine, Anne Perruisseau-Carrier, Corinne Becker, Frédéric Sedel, Luc Téot, Denis Barritault

## Abstract

**Background:**

CACIPLIQ20 (OTR3, Paris, France) is a medical device used for the treatment of chronic skin ulcers. It contains a heparan sulfate mimetic that accelerates tissue healing by stabilizing matrix proteins and protecting heparin-binding growth factors. In humans, an open self-controlled study suggested that the topical application of CACIPLIQ20 optimizes skin healing following surgery.

**Objectives:**

To expand previous findings using a different CACIPLIQ20 administration regimen.

**Methods:**

Twenty-four females were referred for breast-reduction surgery. Each patient had their own control with 1 CACIPLIQ20-treated and 1 saline-treated control breast. The treated side (right or left) was randomly assigned by the operating surgeon. Scar appearance was assessed by 6 independent raters using a global visual scar comparison scale based on scar photographs. All raters were blinded toward the CACIPLIQ20-treated side.

**Results:**

The follow-up period following surgery ranged from 1 to 12 months with a median follow-up of 6 months. Overall, there was a mean improvement of 15.2% (SD = 26.7) in favor of CACIPLIQ20 (*P* = .016). On the CACIPLIQ20-treated side, the mean score per patient was above 20% in 11 patients and above 30% improvement in 8 cases. In contrast, only 3 patients were considered improved by at least 20% on the control side and only 1 above 30%. A comparison of different application regimens suggested that the best trend was obtained with a single administration of CACIPLIQ20 at Day 0.

**Conclusions:**

In conclusion, CACIPLIQ20 could represent an interesting scar prophylactic therapy, based on a single administration at the time of surgery, and without any known adverse effects.

**Level of Evidence: 3:**

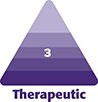

Cutaneous scars develop after surgical procedures or injury, due to the production of collagen-rich connective tissue. Typically, scar matures after a few weeks, becoming lighter and narrower, although full maturation can take up to 2 years.^1,2^ Scar symptoms encompass redness, itching, pain, thickness of the skin, pigmentation, and contractures. Cosmetic scars, especially those that appear after invasive surgical procedures, can cause psychological distress. Furthermore, an abnormal scarring process can result in hypertrophic scars or keloids. Preventing pathological scarring should ideally be started as early as possible after the injury or surgery.^2,3^ Good surgical technique is essential to minimize scarring, but currently there are no recommended prophylactic pharmaceuticals or medical devices that could be administered during surgery to supplement surgical skill.^4,5^

Scars are composed of derma-like collagens covered by the epidermis. The sequence of tissue repair after injury is a tightly regulated process. Initial platelet aggregation and provisional matrix deposition are followed by the influx of inflammatory cells and subsequent cell proliferation, leading to fibroplasia and angiogenesis. Collagen deposition starts within 3 days, reaching its peak in the first few weeks with a combination of Type 1 and Type 3 collagens followed by increased Type 1 collagen that accompanies matrix organization and scar strength.^3,6^

CACIPLIQ20 (OTR3, Paris, France) is a medical device used for the treatment of chronic skin ulcers and contains OTR4120, a biodegradable polymer that mimics the action of endogenous heparan sulfates (HSs) present in the extracellular matrix (ECM). OTR4120 replaces degraded endogenous HS and consequently restores the ECM scaffold by direct physical interactions with fibrous proteins (including collagens and glycoproteins such as fibronectins and laminins). The secondary action of OTR4120 consists in the protection of communication peptides and of matrix elements: the restored scaffold decreases access to proteases by steric hindrance.^7^

The tissue protection and repair functionalities of matrix-regenerating agents (RGTAs) have been tested so far in as many as 20 different tissue injury models in 7 animal species.^7^ In addition to their action on chronic wounds, RGTAs were found to improve speed and quality of healing after acute surgical, traumatic, or ischemic skin lesions and burns in several in vivo animal models.^8–14^ In these studies, healing quality was assessed by measuring the Collagens 1 and 3 neosynthesis, and their ratio showing that RGTA treatment induced a reduction of Collagen 3 synthesis, lower fibrotic index, scar reduction, higher resistance to breakage forces, and improved histology. In humans, an open self-controlled study by Zakine and Le Louarn^15^ suggested that the topical application of OTR4120 (CACIPLIQ20) optimizes skin healing and vascularization in patients following surgery. The purpose of this publication was to expand the initial findings in another cohort of 24 females with mammoplasties using a different CACIPLIQ20 administration regimen and performing a blind review by a panel of 7 raters.

## METHODS

Between 2008 and 2010, 24 patients operated by the same surgeon (G.Z.) for mammoplasty were voluntary to receive CACIPLIQ20 unilaterally. All patients had signed an informed consent form. Written consent was provided by which the patients agreed to the use and analysis of their data. Although a formal institutional review board process was not available, the principles outlined in the Declaration of Helsinki have been followed. The same surgical technique, reductive breast surgery based on the superior pedicle technique, without cutaneoglandular cleavage, with “boat keel” resection and scars in “marine anchor” was performed in cases of breast hypertrophy. Closure was performed under aspirative drainage by deep dermal and superficial intradermal running sutures using colorless, absorbable Polysorb 3/0 (Covidien, Dublin, Ireland). Knots blocking the superficial polysorb running sutures were removed on Day 15, in accordance with standard procedures.

Each patient had their own control with 1 CACIPLIQ20-treated and 1 saline-treated control breast. The treated side (right or left) was randomly assigned by the surgeon (G.Z.). Scar photographs were taken by the same surgeon (G.Z.), with the same lighting and same camera.

Patients included in the present study were not previously reported in the article by Zakine and Le Louarn^15^ where a different treatment protocol was used (applications at Days 1, 4, 8, and 11). It was only recently that the first author decided to reanalyze his database collected in 2008-2010 with a new methodology that could serve as the basis for a prospective randomized controlled trial. The current study had 2 goals: (1) to perform a blinded review of scar photographs by a panel of raters, and (2) to use this analysis to depict the best treatment regimen that could be selected for a prospective double-blind randomized controlled trial.

In the present study, several treatment regimens were tried. For each patient, the treatment regimen is detailed in Table: In most cases (*n* = 14), CACIPLIQ20 was applied only once at the end of the surgery (Day 0). Following the bilateral breast reduction, surgical incisions were sutured according to local standard procedures. The areole and the vertical incision of 1 breast received 5 mL of CACIPLIQ20 applied via an imbibed sterile gauze, while the other breast received 5 mL of a saline solution applied with a sterile gauze (control). CACIPLIQ20 was administered according to the manufacturer’s instructions (Supplemental Figure): (1) Open the blister, move the forceps to access the gauze, open the vial, and pour the solution onto the gauze; (2) Carefully impregnate the wound with the soaked gauze. Use forceps to place the gauze on the cleaned wound, unfold if necessary. Leave the gauze on the wound for 5 min; (3) After 5 min, remove the gauze. Cover with a primary dressing according to standard practice. No additional scar therapy was administered to any of the patient.

In 6 patients, subsequent applications of CACIPLIQ20 were performed depending on the follow-up scheduled visits, usually every 3 days or once a week, up to Day 15 at the latest. In 4 patients, applications of CACIPLIQ20 started on Day 1, that is, after the surgery. In such cases, the treatment was usually reapplied every 3 days up to Day 12. The procedure described above for CACIPLIQ20 administration was also used in patients who received treatments after surgery.

The tolerance and the effects on wound healing were evaluated by clinical examinations and scar photographs performed in the context of standard clinical follow-up, usually at 7 days, 15 days, and then every 3 months. Scar appearance was assessed using a global visual scar comparison scale based on scar photographs.^1,16^ The assessment was performed using the latest photographs available following surgery. The scar global comparison scale assesses the difference in appearance of the 2 scars. The fully anonymized photographic records of each scar were placed side by side over a double-ended visual analog scale, which represents percentage scar improvement. The global scar comparison scale indicates by how much one of the scars has a better appearance than the contralateral scar: “0” means that there is no detectable difference between the 2 scars, while “100%” means that 1 particular scar has a maximally better appearance than the contralateral scar to a level where the improved scar is indistinguishable from the normal skin. Evaluation of global scar appearance was performed by 6 independent raters based on anonymized pictures of scars. All raters were blinded toward the CACIPLIQ20-treated side. Two raters (1 and 2, L.T. and A.P.C., respectively) are plastic surgeons with strong expertise in scar assessment; 2 raters (3 and 4) are physicians and employees of OTR3 with no specific expertise in scar assessment; and 2 raters (5 and 6) are nonphysicians (engineers/scientists) and employees of OTR3. Blinded raters were asked to focus their overall evaluation of scars on the areole and the vertical suture. After deblinding, values favoring the treated breast were transformed into positive values, while values favoring the control breast were transformed into negative values.

A Wilcoxon signed rank test was applied to compare the mean of the global comparison scale calculated from the 6 raters’ evaluations with the null hypothesis of a null median. The analysis was performed on the whole population, combining all treatment regimens. Subgroup analyses were also performed to identify trends in subgroups of patients who received different treatment regimens. Subgroup analyses were descriptive and did not include statistical tests.

Intraclass correlation coefficient (ICC) was calculated with R software using a 2-way random-effect model. A separate, seventh blinded rater (C.B., Rater 7), who is a plastic surgeon specialized in scar assessment, performed an independent evaluation of the scar based on her clinical global impression. This rater was asked if 1 of the 2 scars had a better appearance or not.

## RESULTS

Twenty-four females were included in this retrospective study. Their ages ranged from 16 to 57 years (mean 36.6). Table summarizes which side was treated with CACIPLIQ20 and the treatment regimen.

**Table.** Clinical Characteristics, Treatments Regimen, and Mean Results from Blinded Evaluations

Patient no.	CACIPLIQ20 administration	Treated side (R/L)	Latest assessment (months)	Global comparison scale (mean values per patient)	SD	Remarks
1	D0	R	12	67	10	No comment
2	D0	R	12	40	11	No comment
3	D0	L	1.4	44	21	D21: right breast showing purulent discharge
4	DO, D4, D7	R	6	25	10	D7: less inflammation on the right side, at month 2: palpation is less painful on the right
5	D1, D4, D8, D12	R	6	0	39	D15: more pain on the right
6	D0	R	12	52	12	D15: edema on the left side, D21: pruritus on the left side nothing on the right side, 5 weeks: pruritus on the left
7	D1, D4, D7	R	6	−10	15	D7: less inflammation on the treated side
8	D1, D4	L	1.7	48	21	D15: more pain on the right, no pain on the left
9	D0	R	12	11	9	D30: pruritus on the left
10	D0, D3, D8	R	6	−12	13	D8: no difference
11	D0	L	3	−20	0	Pruritus on the right after month 1
12	D0	R	9	−3	29	No comment
13	D0, D8, D11	R	1.7	41	14	J15: no difference, M1: the control breasts is more inflammatory, indurated and painful
14	D0	R	6	38	15	D15: no difference in pain, control side with slightly more inflammation, D21: a little bit more inflammation on the control side
15	D0, D8, D11	L	9	7	22	No comment
16	D0	R	20	32	20	D45: more pruritus on the left
17	D0	R	6	5	11	No comment
18	DO	R	3	−14	17	No comment
19	D0, D8, D11	R	3	23	14	No difference at month 1
20	D0, D7, D15	R	3	0	24	No comment
21	D1, D3, D6	R	1	28	8	No comment
22	D0	L	12	−32	16	No comment
23	D0	R	3	18	17	D8: no difference
24	D0	R	12	−23	17	No comment
Mean	NA	NA	7.7	15.2	NA	NA
SD	NA	NA	4.7	26.7	NA	NA

L, left; NA, not applicable; R, right; SD, standard deviation.

In most cases (*n* = 14), CACIPLIQ20 was applied only once at the end of the surgery. In 6 patients, subsequent applications were performed depending on the follow-up scheduled visits, usually every 3 days or once a week, up to Day 15 at the latest. In 4 patients, applications of CACIPLIQ20 started on Day 1, that is, after the surgery. In such cases, the treatment was usually reapplied every 3 days up to Day 12. The follow-up period following surgery ranged from 1 to 12 months with a median follow-up of 6 months and a mean of 7.7 months.

The clinical notes of the surgeon are reported in Table. Written information was available in 14 cases (for the other cases, the information was missing). Adverse events were reported in few patients including signs of inflammation or infection (purulent discharge in 1 case). However, these symptoms were considered part of the surgical procedure and were never attributed to CACIPLIQ20 administration. In the majority of cases for whom information was available (10/14), there was an improvement of scar symptoms on the treated side, including inflammation (*n* = 6), pruritus (*n* = 4), or pain (*n* = 2). In 3 cases, no differences were noticed, and in Case 5, more pain was noticed on the treated side.

Results from the global comparison scale performed by 6 independent blinded raters are displayed in Table and Supplemental Table, and 8 selected examples are presented in the Figure. In general, there was a good agreement between raters regarding the notations. We calculated the ICC in order to assess the agreement between raters. It was estimated at 0.65 (*P* < .0001; 95% confidence interval = .493 < ICC < .798). Overall, by combining the scores from the 6 raters, there was a mean improvement of 15.2% (SD = 26.7) in favor of CACIPLIQ20, (*P* = .016, Wilcoxon signed rank test). The mean scores per rater were all positive ranging from 12.1% to 18.8%. The evaluations from specialized plastic surgeons (Raters 1 and 2) were overall consistent with those from 2 nonspecialized physicians (Raters 3 and 4) and with those from nonphysicians (Raters 5 and 6). On the CACIPLIQ20-treated side, the mean score per patient obtained from the 6 individual raters was 20% improvement or above in 11 patients (Cases 1, 2, 3, 4, 6, 8, 13, 14, 16, 19, and 21) and above 30% improvement in 8 cases (Cases 1, 2, 3, 6, 8, 13, 14, and 16). In contrast, only 3 patients were considered improved by at least 20% on average on the control side (Cases 11, 22, and 24) and only 1 (Case 22) above 30%.

**Figure.**
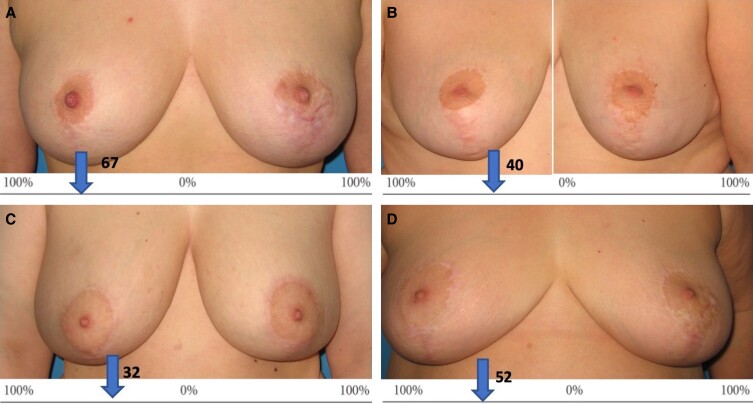
Examples of evaluations using the global comparison scale. This scale indicates by how much one of the scars has a better appearance than the contralateral scar: “0” means that there is no detectable difference between the 2 scars, while “100%” means that 1 particular scar has a maximally better appearance than the contralateral scar to a level where the improved scar is indistinguishable from the normal skin. Assessments focused on the areole and vertical scars. Mean scores per patients are displayed on the CACIPLIQ20 (OTR3, Paris, France)-treated sides. (A) A 42-year-old female patient, improved by an average of 67% at 12 months. (B) A 36-year-old female patient, improved by an average of 40% at 12 months. (C) A 33-year-old female patient, improved by an average of 32% at 20 months. (D) A 53-year-old female patient, improved by an average of 52% at 12 months.

Among the 4 patients who had been treated with a single administration of CACIPLIQ20 at Day 0, 6 (42%) were considered improved by an average score of at least 30%, and the average score in this group was +15.4 (SD = 30.8). Among the 6 patients who received multiple administrations of CACIPLIQ20 starting on Day 0, 1 (17%) was considered improved by an average score of at least 30% and the mean score in this group was +14 (SD = 19.2). In the group of 4 patients who received CACIPLIQ20 starting at Day 1, only 1 patient was considered improved above 30% (25%) and the mean score in this group was +16.7 (SD = 26.6).

One additional rater (Rater 7) performed her own blinded global evaluation without using the global comparison scale. After deblinding, this rater found a better overall outcome on the CACIPLIQ20 side in 12 cases, no difference in 8 cases, and a better appearance on the control side in 4 cases. In most cases, the global impression of this rater was consistent with the mean evaluation of the 6 raters who filled the global comparison scale except 1 divergent evaluation in Patient 11 (Table).

## DISCUSSION

In this self-controlled, retrospective case study, we assessed the effects of CACIPLIQ20 on scar prevention by comparing the effects of CACIPLIQ20 administration on 1 breast to those the other breast, which was treated with saline solution and used as a control. No adverse effects were reported in association with CACIPLIQ20 administration.

In a first study by Zakine and Le Louarn,^15^ a group of 17 patients who underwent mammoplasty for breast hypertrophy were treated by topical cutaneous application of CACIPLIQ20 on 1 breast, while the opposite control breast was treated in a similar manner with physiological saline solution (the diluent for CACIPLIQ20). Treatment consisted of 4 topical applications of CACIPLIQ20, using a nontissue sterile compress impregnated with CACIPLIQ20 solution, applied to vertical and peri-areolar wounds for 5 min, at Days 1, 4, 8, and 11. The evaluation was based on the tolerance of the product and the quality of healing, as determined by photographic and clinical examinations on Days 8, 15, and 30 and after 3 months. No adverse effects were reported. Inflammation, pruritis, and hypertrophic scars were less frequent on the side treated with CACIPLIQ20. After 3 months, the average Vancouver score was 5.18 ± 1.20 in the treated group and 5.71 ± 1.30 in the control-treated group. Although these results were encouraging, analyses were performed by a single investigator who was unblinded regarding the treatment side. Furthermore, only 1 treatment regimen was evaluated.

Our analyses of the 24 additional cases presented in this publication tend to confirm the initial results. In this new study, assessments were performed blindly by 6 independent raters, based on anonymized digitized images, using a global comparison analogic scale. Results showed an overall better outcome of scars on the CACIPLIQ20-treated side of +15.2% (*P* = .016). Although scar appearance was statistically better on the CACIPLIQ20-treated side, in a minority of patients, we observed a better scar outcome on the control side. These observations can be explained by intrapatient asymmetry in the wound healing process. Such asymmetry could have been caused by several local confounding factors such as wound tension, local hypoxia, or inflammatory reaction to suture material.

The ICC was estimated at 0.65 (*P* < .0001) showing a highly significant, albeit moderate agreement between raters. Such variability among raters is not surprising given the purely subjective nature of the scale. Of note, a comparison of different application regimens suggested that the best trend was obtained with a single administration of CACIPLIQ20 at Day 0 and that further applications or later treatments starting at Day 1 did not provide additional positive effects.

CACIPLIQ20 mimics HSs, a class of matrix glycosaminoglycans associated with tissue regeneration through the binding of endogenous structural proteins, communication peptides, or growth factors. OTR4120, the main component of CACIPLIQ20 was shown to improve both speed and quality of wound healing in preclinical models.^8–14^ From these previous experiments, OTR4120 noticeably increased mechanical wound breaking strength, improved microcirculation, reduced inflammation, accelerated the maturation of epidermal structures and granulation tissue formation, restored near-normal Collagen I/Collagen III expressions, increased expression of endothelial growth factor, platelet-derived growth factor, and transforming growth factor beta-1.

OTR4120 has also showed clinical benefits in many pathological conditions, including corneal neurotrophic ulcers, corneal ulcers caused by viral infections, corneal dystrophy, keratoconus surgery, superficial ulcerative keratitis of Sjogren's syndrome, nonhealing chronic skin ulcers, cutaneous manifestations of sickle cell disease, epidermolysis bullosa, mechanical, and burn injuries.^7,17^

Our results suggest that CACIPLIQ20 administration the day of surgery has a long-term effect on improving subsequent scar formation. International recommendations do not recommend any products or devices to be administered systematically to patients at the time of surgery to prevent unsightly or pathological scarring.^4,5^ For high-risk wounds, silicone-based products are the preferred preventative measure. Silicone gel or sheeting is usually applied after the wound has epithelialized. Some silicone-based wound dressings such as “Stratamed” can also be used just after surgery, to treat sutured wounds and prevent abnormal scarring. Options for patients at lower but still elevated risk include silicone gel or sheeting, hypoallergenic microporous tape, or onion extract–containing preparations. Hopes are that through earlier intervention in fresh wounds or fresh scars, excessive scarring could be limited or prevented. The development of such scar prophylactic agents is an active research area. Several potential scar-preventing products targeting growth factors involved in the cutaneous wound healing process have been tested in clinical trials, including recombinant TGFb3, interleukin 10, siRNA and antisense oligonucleotides that downregulate the expression of connective tissue growth factor.^18^ However, none of these products has conclusively demonstrated efficacy in human trials so far, and none has been approved to date. A preventive treatment for scars was developed with the laser-assisted skin healing (LASH), using an 810 nm laser diode. Applied immediately after surgery, this device activates tissue regeneration through the overexpression of heat shock protein 70 which in turn is believed to hasten scar maturation.^19^ A double-blind, controlled, randomized clinical trial, assessing the efficacy of the device after mammoplasty resulted in a better overall appearance of scars for the laser-treated scars when compared with the control group.^20^ The LASH technology is currently commercialized as “Urgotouch” (Urgo, Chenôve, France). Several randomized controlled trials with botulinum toxin A demonstrated that injections administered after wound closure enhanced wound healing and less noticeable scars compared to placebo.^21–23^ Recombinant human basic fibroblast growth factor (FGF) is marketed in Japan as a topical spray for accelerating the healing of burn wounds and diabetic leg ulcers. Basic FGF is currently recommended in Japan for the treatment of second-degree burns.^24^ In Europe, Episalvan (topical betulin gel [TBG]; European Medicines Agency, Amsterdam, the Netherlands) is approved for the treatment of partial thickness wounds (ie, wounds that affect the superficial layers of the skin, including epidermis and dermis). Two randomized, controlled, multicenter Phase III clinical trials concluded that TBG accelerates re-epithelialization of partial thickness wounds compared to the current standard of care.^25^

Our study has methodological limitations. First, it is based on a relatively small number of patients. Although the main statistical analysis performed on the whole population shows a statistical effect favoring CACIPLIQ20, the subgroup analyses that aim to depict which treatment regimen seems to work the best rely on descriptive analyses. The low numbers of patients in each category do not allow robust statistical analyses and these subgroup analyses can only identify trends. Second, although the evaluations were performed blindly by independent raters, the study is based on retrospective assessments. Third, the follow-up was 1 to 12 months—which might be considered too short in some patients to assess complete healing. However, 20 patients (20/24) had a follow-up of at least 3 months (and 15/24 of at least 6 months) which can be considered adequate to assess the scar formation process.

Although this retrospective study is based on a re-analysis of photographic data that were acquired about 10 years ago, the new analyses are robust and provide important insights ahead of a prospective double-blind, placebo, controlled trial that is underway.^26^

## CONCLUSIONS

CACIPLIQ20 could represent an interesting scar prophylactic therapy, based on a single administration at the time of surgery and without any known adverse effects. These promising results need to be further confirmed in a well-designed, larger prospective double-blind, placebo-controlled randomized clinical trial which has already began.

## Supplementary Material

ojad031_Supplementary_Data
