# Supplementary material for: A Retrospective Self-Controlled Study Evaluating the Prophylactic Effects of CACIPLIQ20 on Postsurgical Scars
Source: Aesthet Surg J Open Forum. 2023 Mar 23;5:ojad031. doi: 10.1093/asjof/ojad031 (PMC10084089; doi:10.1093/asjof/ojad031)
Supplement: ojad031_Supplementary_Data [file ojad031_supplementary_data.zip › 23-0016_Supplemental Table.docx]

| Patient ID | Rater #1 evaluation | Rater #2 evaluation | Rater #3 evaluation | Rater #4 evaluation | Rater #5 evaluation | Rater #6 evaluation | Rater #7 evaluation | Mean values per patient | SD |
| --- | --- | --- | --- | --- | --- | --- | --- | --- | --- |
| 1 | 80 | 80 | 60 | 60 | 60 | 60 | Cacipliq (OTR3, Paris, France) better | 67 | 10 |
| 2 | 40 | 60 | 30 | 30 | 40 | 40 | Cacipliq better | 40 | 11 |
| 3 | 35 | 80 | 50 | 50 | 30 | 20 | Control better | 44 | 21 |
| 4 | 20 | 30 | 10 | 30 | 20 | 40 | Cacipliq better | 25 | 10 |
| 5 | 10 | -60 | 40 | 0 | -30 | 40 | Control better | 0 | 39 |
| 6 | 50 | 60 | 50 | 70 | 40 | 40 | Cacipliq better | 52 | 12 |
| 7 | 0 | -20 | 10 | -20 | 0 | -30 | No difference | -10 | 15 |
| 8 | 50 | 60 | 50 | 80 | 30 | 20 | Cacipliq better | 48 | 21 |
| 9 | 10 | 20 | 0 | 0 | 15 | 20 | No difference | 11 | 9 |
| 10 | 0 | -30 | 0 | -20 | 0 | -20 | Control better | -12 | 13 |
| 11 | -20 | -20 | -20 | -20 | -20 | -20 | Cacipliq better | -20 | 0 |
| 12 | 0 | 40 | 0 | -30 | 15 | -40 | No difference | -3 | 29 |
| 13 | 50 | 50 | 25 | 60 | 30 | 30 | Cacipliq better | 41 | 14 |
| 14 | 25 | 30 | 20 | 60 | 40 | 50 | Cacipliq better | 38 | 15 |
| 15 | 0 | -30 | 30 | 20 | 0 | 20 | No difference | 7 | 22 |
| 16 | 20 | 70 | 20 | 40 | 20 | 20 | Cacipliq better | 32 | 20 |
| 17 | 5 | 0 | -10 | 20 | 15 | 0 | Cacipliq better | 5 | 11 |
| 18 | -30 | -30 | 0 | -30 | 5 | 0 | Control better | -14 | 17 |
| 19 | 0 | 40 | 30 | 20 | 20 | 30 | Cacipliq better | 23 | 14 |
| 20 | 5 | 40 | 0 | -20 | 5 | -30 | No difference | 0 | 24 |
| 21 | 20 | 40 | 30 | 20 | 30 | 30 | Cacipliq better | 28 | 8 |
| 22 | -10 | -50 | -20 | -50 | -30 | -30 | No difference | -32 | 16 |
| 23 | 0 | 40 | 0 | 30 | 10 | 30 | Cacipliq better | 18 | 17 |
| 24 | -15 | -50 | 0 | -20 | -20 | -30 | Control better | -23 | 17 |
| Mean values per rater | 14,4 | 18,8 | 16,9 | 15,8 | 13,5 | 12,1 |  | 15,2 |  |

**Supplemental Table:** Detailed Results from Blinded Evaluations

Positive values mean that the cacipliq treated side is improved, negative values mean that the control side is improved compared to the contralateral side. SD, standard deviation.
