# Supplementary figures and images for: A Retrospective Self-Controlled Study Evaluating the Prophylactic Effects of CACIPLIQ20 on Postsurgical Scars
Source: Aesthet Surg J Open Forum. 2023 Mar 23;5:ojad031. doi: 10.1093/asjof/ojad031 (PMC10084089; doi:10.1093/asjof/ojad031)

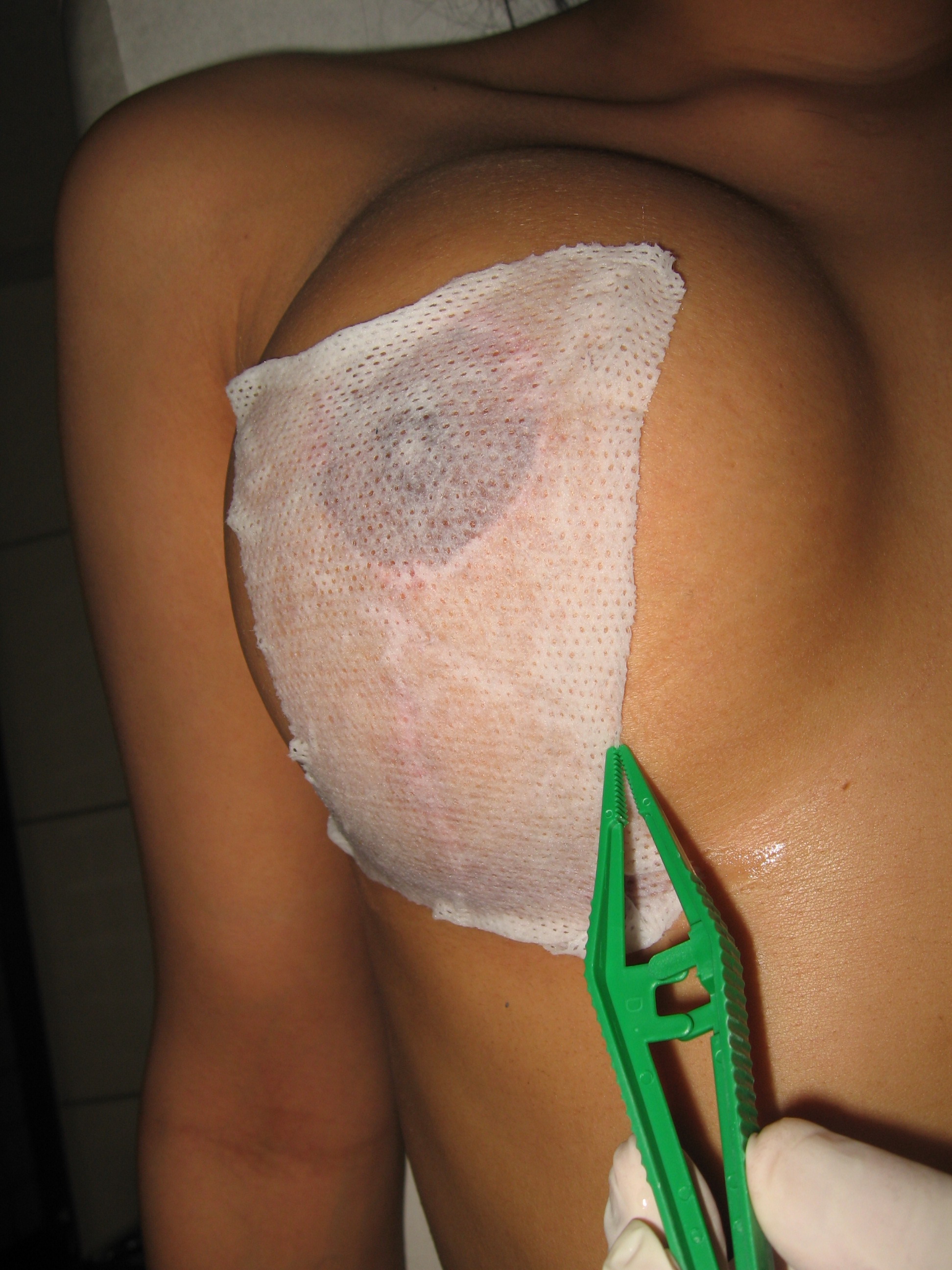

Supplement: ojad031_Supplementary_Data [file ojad031_supplementary_data.zip › 23-0016_Supplemental Figure.JPG]
